# Prescribers’ Knowledge, Attitudes and Behaviors on Antibiotics, Antibiotic Use and Antibiotic Resistance in Jordan

**DOI:** 10.3390/antibiotics10070858

**Published:** 2021-07-15

**Authors:** Reema A. Karasneh, Sayer I. Al-Azzam, Mera Ababneh, Ola Al-Azzeh, Ola B. Al-Batayneh, Suhaib M. Muflih, Mohammad Khasawneh, Abdo-Rahman M. Khassawneh, Yousef S. Khader, Barbara R. Conway, Mamoon A. Aldeyab

**Affiliations:** 1 Department of Basic Medical Sciences, Faculty of Medicine, Yarmouk University, Irbid 21163, Jordan; reema.karasneh@yu.edu.jo; 2 Department of Clinical Pharmacy, Faculty of Pharmacy, Jordan University of Science and Technology, Irbid 22110, Jordan; salazzam@just.edu.jo (S.I.A.-A.); mababneh@just.edu.jo (M.A.); smmuflih@just.edu.jo (S.M.M.); 3 Department of Pharmacy Practice, College of Pharmacy, King Saud Bin Abdulaziz University for Health Sciences, Riyadh 11481, Saudi Arabia; azzeho@ksau-hs.edu.sa; 4 Department of Preventive Dentistry, Faculty of Dentistry, Jordan University of Science and Technology, Irbid 22110, Jordan; olabt@just.edu.jo; 5 Department of Pediatrics and Neonatology, Faculty of Medicine, Jordan University of Science and Technology, Irbid 22110, Jordan; deema321@just.edu.jo; 6 Faculty of Medicine, Jordan University of Science and Technology, Irbid 22110, Jordan; aboodkhassawneh62@gmail.com; 7 Department of Public Health, Faculty of Medicine, Jordan University of Science and Technology, Irbid 22110, Jordan; yskhader@just.edu.jo; 8 Department of Pharmacy, School of Applied Sciences, University of Huddersfield, Huddersfield HD13DH, UK; b.r.conway@hud.ac.uk; 9 Institute of Skin Integrity and Infection Prevention, University of Huddersfield, Huddersfield HD13DH, UK

**Keywords:** attitudes, prescribers, antibiotics, physicians, dentists, antibiotics resistance

## Abstract

More research is needed on the drivers of irrational antibiotic prescribing among healthcare professionals and to ensure effective prescribing and an adequate understanding of the issue of antibiotic resistance. This study aimed at evaluating prescribers’ knowledge, attitudes and behaviors about antibiotic use and antibiotic resistance. A cross-sectional study was conducted utilizing an online questionnaire and included physicians and dentists from all sectors in Jordan. A total of 613 prescribers were included (physicians *n* = 409, dentists *n* = 204). Respondents’ knowledge on effective use, unnecessary use or associated side effects of antibiotics was high (>90%), compared with their knowledge on the spread of antibiotic resistance (62.2%). For ease of access to the required guidelines on managing infections, and to materials that advise on prudent antibiotic use and antibiotic resistance, prescribers agreed in 62% and 46.1% of cases, respectively. 28.4% of respondents had prescribed antibiotics when they would have preferred not to do so more than once a day or more than once a week. Among respondents who prescribed antibiotics, 63.4% would never or rarely give out resources on prudent use of antibiotics for infections. The findings are of importance to inform antibiotic stewardships about relevant interventions aimed at changing prescribers’ behaviors and improving antibiotic prescribing practices.

## 1. Introduction

Antimicrobial resistance (AMR) poses a significant problem to public health that threatens the provision of effective treatment for a range of infections and undermines the efforts to control the development and spread of multidrug-resistant (MDR) bacteria [1]. The development of AMR has been shown to lead to longer hospital stays, increased mortality and higher healthcare costs [1,2,3]. A review estimating the global economic cost of antimicrobial resistance, the O’Neill report, revealed that a continued rise in resistance would lead to 10 million people dying every year by 2050, resulting in projected costs as high as USD 100 trillion worldwide [2]. It is anticipated that the economic impact of AMR will be greatest in developing countries, leading to increasing economic inequality between countries [4]. In addition, maintaining antibiotic effectiveness is critical for the sustainability of modern healthcare systems. Common infections will be associated with more severe complications and an increased risk of death, and several fundamental healthcare processes could be unavailable or unsafe [5]. Therefore, actions are urgently needed to minimize the emergence of AMR. The link between antibiotic use and the development and spread of bacterial resistance has been demonstrated in several studies [1,2,6,7,8,9]. Studies suggest that between 25% and 50% of hospitalized patients receive antibiotics, of which approximately 20–50% of all antimicrobials prescribed are either unnecessary or inappropriately used [10,11,12,13]. The impact of the rapid evolution of resistance on reducing the potential for sustained efficacy of current classes of antibiotics, or newly developed antibiotics, highlights the importance of promoting appropriate antimicrobial prescribing practices [14,15]. In response to the growing threat of antimicrobial resistance, the World Health Assembly adopted a global action plan (GAP) on AMR [16].

Antibiotic prescribing is a complex process influenced by several factors including the healthcare system, physicians, other healthcare providers, patients and the general public [17]. A lack of understanding and clarity about prudent antimicrobial use, and the spread of antimicrobial-resistant microorganisms present important barriers to the prevention and control of AMR [18,19,20]. Since changes in antibiotic prescribing patterns will need changes in healthcare professionals’ behavior, it is necessary to understand the key factors influencing their prescribing and what interventions may effect change. Therefore, the aim of this study was to evaluate prescribers’ knowledge, attitudes and behaviors regarding antibiotics, antibiotic use and antibiotic resistance in Jordan, as one of the Middle East countries. Reports form Jordanian hospitals showed a high prevalence of AMR, with particular note to methicillin-resistant Staphylococcus aureus (MRSA) that reached a rate of 45% [21].

## 2. Results

### Prescribers’ Sociodemographic Characterestics and Actual Knowledge

Table 1 shows the characteristics of the respondents, with 71.0% being in the age group of 24–35 years and more, and 64.1% of those participating in the study being male. Two thirds (66.7%) of the study participants were medical doctors (physicians), and around half of the participants were generalists (49.8%) and providing care in a hospital setting (46.5%).

More than 90% of the respondents correctly answered the questions on effective use, unnecessary use or associated side effects of antibiotics; however, lower percentages of correct answers were observed for questions on the spread of antibiotic resistance (Table 2).

Knowledge average scores were found to be significantly higher among physicians (*p* = 0.0031), middle-age groups (36–55 years) (*p* = 0.0140) and specialists (*p* < 0.0001) (Table 3). Higher proportions of full scores were also found to be significantly higher among physicians (*p* = 0.0350), and more years of experience (*p* = 0.0183), as shown in Table 3.

Further, a multivariable stepwise regression model was conducted, and only profession and age were found to be statistically significant predictors of higher knowledge scores. The results of the univariate analysis are presented in Table 4. Physicians and respondents’ score in the age group of 36–55 years had higher knowledge (estimate: 0.24, CI: 0.12–0.34, *p* < 0.0001; estimate: 0.27, CI: 0.09–0.37, *p* = 0.0152, respectively).

The results of perceived knowledge (Table 5) show that most of the respondents either agree or strongly agree that they know what antibiotic resistance is and what information to give to individuals about prudent use of antibiotics and antibiotic resistance, and that they have sufficient knowledge about how to use antibiotics appropriately (87.11%, 81.56% and 85.63%, respectively) (Table 5).

Around two thirds of the prescribers agreed or strongly agreed that they have easy access to the required guidelines on managing infections (62.0%), and around half agreed or strongly agreed that they have easy access to materials that advise on prudent antibiotic use and antibiotic resistance (46.1%). Moreover, 67.3% agreed or strongly agreed they had a good opportunity to provide such advice (Table 5). Nevertheless, 42.1% of prescribers would advise on prudent use of antibiotics for infections more than once a day or a week (Table 6).

The majority of prescribers (85.6%) agreed or strongly agreed that they would consider antibiotic resistance when treating a patient. Around 75% of prescribers were confident in making prescribing decisions and confident about the available antibiotics guidelines on managing infections (Table 5). In addition, two thirds of prescribers agreed or strongly agreed that there is a connection between their prescription and antibiotic resistance, that they had a key role in helping control antibiotic resistance and that they felt supported not to prescribe antibiotics when they are not necessary. Several strategies were employed to prescribe antibiotics prudently including delayed prescribing (*n* = 210, 34.3%), patient education (*n* = 422, 68.8%) and new patient consultation (*n* = 90, 14.7%). However, 73 (11.9%) prescribers would not follow any strategy.

Most of the respondents would prescribe antibiotics daily (*n* = 262, 42.7%) or weekly (*n* = 209, 34.1), with 10.8% (*n* = 66) monthly, 6.0% (*n* = 37) quarterly and 6.4% (*n* = 39) yearly. Although 27.6% of prescribers did prescribe antibiotics more than once a day, more than 50% would never or rarely give out resources on prudent use of antibiotics for infections (Table 6). Reasons for not providing resources included the following: “Patient does not require information” (*n* = 73, 11.9%), “Patient uninterested in information” (*n* = 244, 39.8%%), “Insufficient time” (*n* = 183, 29.9%), “Difficulty getting patient to understand diagnosis” (*n* = 139, 22.7%), “Language barriers” (*n* = 17, 2.8%), “No resources available” (*n* = 162, 12.0%), “I was not sure what advice to provide” (*n* = 28, 4.6%) and “I was able to give out advice or resources as needed” (*n* = 185, 30.2%).

Regarding behaviors on drivers to initiate prescriptions, 47.2% of respondents would rarely or never prescribe antibiotics because of fear of patient deterioration or fear of complications. On the other hand, 28.4% of respondents have prescribed antibiotics when they have preferred not to do so more than once a day or more than once a week, and 10.3% of respondents had prescribed antibiotics once a week because it took less time than to explain the reason why they are not indicated during the last week. Two thirds of respondents claimed that they would never or rarely prescribe antibiotics in situations in which it is impossible to conduct a follow-up of the patient or maintain a relationship with the patient, or due to being uncertain about the diagnosis of infection. Around 40% of respondents would never stop an antibiotic prescription earlier than the prescribed course length, prescribe a shorter course of treatment compared to available guidelines or discontinue early treatment because a bacterial infection was not likely after all.

Concerning the One Health question, most of the respondents were unsure if it is legal to use antibiotics to stimulate growth in farm animals, with only 14.2% answering correctly (Table 2). Furthermore, 59.8% of participants agreed or strongly agreed that excessive use of antibiotics in livestock and food production is important in contributing to antibiotic resistance in bacteria from humans. In comparison, 40.9% (*n* = 251) of respondents could not decide whether environmental factors contribute to antibiotic resistance (Table 5). When asked about the level of when it is most effective to tackle resistance to antibiotics, only 55.3% (*n* = 339) answered that action at all levels is required. Others responded at the individual level (prescribers) (*n* = 230, 37.5%), regional/national level (*n* = 128, 20.9%), global level (*n* = 120, 19.6%) and environmental or animal health level (*n* = 31, 5.1%), and 6.0% (*n* = 37) did not know the answer.

## 3. Discussion

To our knowledge, this is the first study in the Middle East area to assess the knowledge, attitudes and behaviors about antibiotic use and antibiotic resistance among physicians and dentists using the ECDC’s validated instrument [22,23]. Antimicrobial resistance is an emerging health threat worldwide, driving a real necessity for rational antibiotic use and following guidelines in treating patients to preserve antimicrobial activity [24,25]. In our study, physicians’ knowledge regarding antibiotic resistance was found to be higher compared to dentists and was significantly linked to more years in practice. Most responders in the studied group believed that they have adequate knowledge about antimicrobial resistance and the ability to give the right prescription and appropriate medical advice with respect to antibiotics. Although only fifty percent of the prescribers reported that they have access to the most recent guidelines, the majority still believe that they can use antibiotics prudently.

A study conducted by Wushouer and colleagues in China reported that the higher the knowledge of the practitioners, the lower the rate of antibiotic prescribing [26]. This was also reported in a German study that assessed the effect of knowledge about antibiotic resistance on the prescribing pattern among practitioners; awareness about antimicrobial resistance highly influenced the practitioners’ pattern in prescribing antibiotics to their patients, and this was confirmed in additional studies in Iran and Croatia [27,28,29]. Conversely, two studies in Cambodia and Nigeria found that physicians were alert about the challenge related to the misuse of antibiotics, but this did not influence their practice in managing antibiotic prescribing [30,31]. Understanding the factors associated with overprescribing of antibiotics including prescribers’ knowledge is crucial to find strategies to overcome this challenge [32,33,34]. In our study, 45.7% of the participants were specialists with higher knowledge scores when compared to generalists. This may contribute to the proper use of antibiotics as per many studies, particularly as general physicians tend to prescribe antibiotics more commonly compared to specialists [29,35,36].

Additionally, we found that most of the participating physicians and dentists achieved a high score in questions related to effectiveness, inappropriate use and adverse effects but scored less well in questions addressing antimicrobial resistance. This is similar to a study conducted in the United States in which the authors compared the prescribing behavior of those prescribing high rates versus those prescribing low rates of antibiotics [37]. Higher prescribing rates were found to be associated with patient satisfaction and needs rather than antibiotic side effects or evidence-based medicine [37]. Although the requirement for antibiotics is less common in dentistry, there are still many more antibiotic prescriptions which may be attributed to the level of knowledge about antibiotics and antimicrobial resistance among dentists compared to physicians, as observed in our study [38,39]. Half of our participants believed that there is a lack of open access resources to find recent guidelines and information that support their prudent antibiotics use, while a study in Germany found that only 7% of physicians believed this to be true [27]. A Swedish study found that increasing awareness and continuous education for healthcare providers are fundamental to manage antimicrobial resistance [40]. Thus, as with many other countries, provision of continuing education for healthcare providers is required nationally [1,40,41,42].

Multiple factors were found to contribute to antibiotic prescribing decisions including psychological and socioeconomical factors, in addition to clinical aspects [37,43,44,45]. Machowska and colleagues reported that practitioners’ knowledge and attitudes toward antibiotics use, lack of training on antibiotic prescribing during education and pharmaceutical promotional activities were among the most important factors, as well as the physician–patient relationship [44]. Patient satisfaction is another factor that may contribute to antibiotic prescribing decisions, as shown in an Iranian study in which 69% of the patients would be concerned about their medical condition if an antibiotic was not prescribed [28].

Despite the findings that both physicians and dentists knew about strategies to promote prudent antibiotic use, these were not implemented effectively. It is known that delaying antibiotic prescribing can control inappropriate antibiotic use without affecting the health outcomes for patients [46,47]; however, many practitioners in this study did not use this strategy. According to De la Poza Abad and colleagues, 46% of physicians in Spanish primary care settings were found to use this strategy, especially when treating respiratory tract infections [48]. However, our findings indicate that higher percentages of prescribers have used this strategy of delayed prescribing (34.3%) compared to those reported in Germany (29.4%) [27].

The majority of the prescribers indicated that they tend to prescribe antibiotics on a daily basis, similar to high rates reported in other studies [32,47,49]. While the tendency to prescribe antibiotics was high, the practitioners believed that they prescribe antibiotics mostly when indicated. This has not always been the case in other studies, for example, 54% of Cambodian physicians believed that most antibiotic prescriptions were inappropriate [30]. In a nationwide retrospective study for rational use in dentistry, 96.6% of prescriptions were prescribed for irrational indications including dental caries [50]. Furthermore, a qualitative study showed that antibiotics were prescribed due to the fear of deterioration of a patient’s health status [51]. Using antibiotics in poultry is one of the factors that affects antibiotic resistance worldwide [52,53,54]. Lower proportions of participants in our study were aware of the risk of antibiotic resistance related to use in agriculture and food production compared to that reported by the ECDC survey [23].

This study has some limitations. First, this study used an online survey that may introduce selection bias. However, this is less likely to affect our results, particularly as the survey was sent to physicians and dentists (literate population) who are increasingly using the internet and smartphones in their practice [55,56,57]. In addition, our sample included more prescribers of a younger age. This study would benefit from more involvement of prescribers from other age groups. Evidence from the literature suggests that age is less likely to influence the use of smartphones among physicians [58,59].

In conclusion, the results of this study indicate that antibiotic resistance should direct our plans to a more robust system to educate our practitioners and update them about local and global antibiotic use status and antimicrobial resistance. The findings are of importance to inform antimicrobial stewardship with relevant interventions aiming at changing prescribers’ behaviors and improving antibiotic prescribing practices.

## 4. Materials and Methods

### 4.1. Study Design and Setting

A cross-sectional study survey of Jordanian physicians and dentists was conducted using an online questionnaire. This survey was conducted using the European Centre for Disease Prevention and Control’s (ECDC) validated instrument [22,23].

Physicians and dentists from all sectors were eligible to participate in the study. A web-based survey software (Google Forms) was used to collect data, applying the “required” and “Limit to one response” validation options. Physicians and dentists were recruited through a variety of means, including direct mail, online recruiting, online professional conferences and social media. Participants received an invitation leading them to a Google Forms-based online survey with more comprehensive details about the study. Participants were provided with a brief description of the study and informed that their participation was voluntary and that their responses would be anonymized and treated as confidential.

### 4.2. Survey Instrument

A 10 min online questionnaire was adapted from the questionnaire developed and tested by the European Centre for Disease Prevention and Control (ECDC) [22,23] (Questionnaire S1—Supplementary Material). The survey began with questions designed to collect details about sociodemographic variables. The second section asked 8 questions to test respondents’ actual knowledge using true or false questions. Four questions were to assess knowledge on effective use, unnecessary use or associated side effects of antibiotics, three questions were on the spread of antibiotic resistance and one question was to assess knowledge on antibiotic resistance in the context of the animal sector. Scores of this scale ranged between 0 and 8, with one point given for each correct answer.

Section three consisted of a 5-point Likert scale (strongly agree to strongly disagree), in addition to not applicable/I do not understand the question. A level of agreement was obtained on 14 statements, including two statements on antibiotic resistance in the context of food and environmental factors, three statements that assessed perceived knowledge, three statements that assessed opportunities that may affect prudent prescribing and management of infections, three statements that assessed available opportunities to enact positive behaviors for preventing and controlling AMR and six statements that were on prescribers’ motivations to initiate antibiotic prescriptions and antibiotic prescribing decisions, support and accessibility to guidelines.

The fourth section included 12 questions on the frequency with which prescribers provided antibiotics or resources related to the prudent use of antibiotics (3 questions), behavior on drivers for initiating prescriptions (6 questions) and antibiotic prescribing behaviors (3 questions). Questions on the frequency were asked concerning the last week, with options that included more than once a day, more than once a week, once a day, never and rarely, in addition to “not applicable” and “I do not remember” options. The frequency of antibiotic prescribing, in general, was also obtained from respondents. Prescribers were also asked about why they were unable to provide any resources to their patients, strategies employed to prescribe antibiotics prudently and the level of implementation they think would be most effective to tackle resistance to antibiotics.

The questionnaire was administered in both Arabic and English languages for better interpretation. Prescribers had the option to choose any of the two languages. An initial online pretest of the questionnaire was performed with 10 physicians and dentists. Each participant was interviewed to determine whether the questions were correctly interpreted and if the answer options given were adequate. Based on the pretest interviews, minor adjustments were made to the questionnaire. The pilot interview data were not included in the final sample.

### 4.3. Statistical Analysis

The sample size was determined, using Raosoft sample size calculator (http://www.raosoft.com/samplesize.html), based on a margin of error of 5%, confidence level of 95%, population size of 36,000 (total of registered physicians and dentists in Jordan) and a response distribution of 50%. The calculated sample size was 381. Google Forms was connected to a Google spreadsheet for further analysis and data organization. The IBM SPSS (Statistical Package for the Social Sciences, Armonk, NY, USA) version 24.0 software was used to analyze the collected data. The percentages, frequencies, means and standard deviations were used to display the results. An analysis of variance (ANOVA) test or t-test was used to examine the effect of demographic characteristics on respondents’ knowledge scores. The chi-square test or Fisher’s exact test was used to examine differences among respondents with a full knowledge score. Finally, a multivariable linear regression model was conducted to assess predictors of knowledge, adjusting for confounding variables. The significance of all results was determined using a *p*-value of less than 0.05.

## Figures and Tables

**Table 1:** Respondents’ sociodemographic characteristics (*n* = 613).

Variable	Frequency	Percentage %
Gender	Female: 220	35.9%
	Male: 393	64.1%
Age, years	24–35: 435	71.0%
	36–55: 138	22.5%
	≥56: 40	6.5%
Profession	Medical Doctor (physician): 409	66.7%
	Dentist: 204	33.3%
Role of specialist	Specialist: 280	45.7%
	Generalist: 305	49.8%
	Academia/Research: 28	4.6%
Years of practice (*n*)	0–5: 376	61.3%
	6–15: 127	20.7%
	≥ 16: 110	18.0%
Place of practice	Public Clinic: 74	12.1%
	Academia/Research: 45	7.3%
	Hospital: 285	46.5%
	Private Clinic: 209	34.1%
Governorate (region)	North: 288	47.0%
	Middle: 289	47.2%
	South: 36	5.9%

**Table 2:** Respondents’ actual knowledge.

Key Knowledge Questions	Correct Answer	Answer	Percentage %
Antibiotics are effective against viruses:	False	False: 587	95.8%
		True: 16	2.6%
		Unsure: 10	1.6%
Antibiotics are effective against cold infections:	False	False: 559	91.2%
		True: 40	6.5%
		Unsure: 14	2.3%
Unnecessary use of antibiotics makes them become ineffective:	True	False: 33	5.4%
		True: 571	93.1%
		Unsure: 9	1.5%
Taking antibiotics has associated side effects or risks such as diarrhea, colitis, allergies:	True	False: 15	2.4%
		True: 574	93.6%
		Unsure: 24	3.9%
Every person treated with antibiotics is at an increased risk of antibiotic-resistant infection:	True	False: 80	13.1%
		True: 486	79.3%
		Unsure: 47	7.7%
Antibiotic-resistant bacteria can spread from person to person:	True	False: 159	25.9%
		True: 381	62.2%
		Unsure: 73	11.9%
Healthy people can carry antibiotic-resistant bacteria:	True	False: 75	12.2%
		True: 413	67.4%
		Unsure: 125	20.4 %
The use of antibiotics to stimulate growth in farm animals is legal in Jordan:	False	False: 87	14.2%
		True: 86	14.0%
		Unsure: 440	71.8%

**Table 3:** Predictors of actual knowledge scores among prescribers.

Variable		No. of Respondents	Average Score (Range 0–8)	*p*-Value	*n* (%) of Respondents Who Answered Correctly (Full Score)	*p*-Value
Region:	North	288	5.9 ± 1	0.8182	14 (2.3)	0.1839
	Middle	289	6.0 ± 1.2		12 (2.0)	
	South	36	6.0 ± 0.95		0 (0.0)	
Profession:	Physicians	409	6.1 ± 1.2	0.0031	22 (3.6)	0.0350
	Dentists	204	5.7 ± 1.2		4 (0.7)	
Gender:	Female	220	5.9 ± 1.2	0.4818	10 (1.6)	0.7799
	Male	393	5.9 ± 1.2		16 (2.6)	
Age group:	24–35 years	435	5.9 ± 1.2	0.0140	15 (2.4)	0.0185
	36–55 years	138	6.2 ± 1.2		11 (1.8)	
	≥56 years	40	5.9 ± 1.6		-	
Years in practice:	0–5 years	376	5.9 ±1.2	0.0560	11 (1.8)	0.0183
	6–15 years	127	6.0 ± 1.3		5 (0.8)	
	≥16 years	110	6.2 ± 1.2		10 (1.6)	
Role:	Academia/Research	28	5.8 ± 1.6	<0.0001	2 (0.003)	0.0519
	Generalist	305	5.7 ± 1.2		6 (1.0)	
	Specialist	280	6.3 ± 1.1		18 (2.9)	

**Table 4:** Univariate analysis of the relationship between knowledge scores and demographic characteristics of respondents.

Variable	Estimate	95% CI	*p*-Value
Gender			
Female	−0.04	−0.14–0.07	0.49190
Male *			
Region			
Middle	−0.03	−0.2–0.14	0.73557
North	0.03	−0.15–0.20	0.77490
South *			
Profession			
Physician	0.16	0.8–0.25	0.00310
Dentist *			
Age group			
24–35 years	−0.11	−0.28–0.05	0.18090
36–55 years	0.24	0.05–0.18	0.01670
≥56 years *			
Years in practice			
≥16 years	0.18	0.09–0.27	0.04200
6–15 years	−0.03	−0.21–0.11	0.67040
0–5 years *			
Role			
Specialist	0.34	0.15–0.41	0.00140
Academic/Research	−0.13	−0.44–0.18	0.39820
Generalist *			

* Indicates reference variable.

**Table 5:** Perceived knowledge, opportunity and motivation to prescribe antibiotics.

Item	SA	A	D	SD	N/A	U	IDU
One Health: environmental and animal health factors that are important in contributing to antibiotic resistance in bacteria from humans							
Excessive use of antibiotics in livestock and food production is important in contributing to antibiotic resistance in bacteria from humans	59 9.6%	308 50.2%	37 6.0%	8 1.3%	-	169 27.6%	32 5.2%
Environmental factors such as wastewater in the environment are important in contributing to antibiotic resistance in bacteria from humans	14 2.3%	180 29.4%	76 12.4%	8 1.3%	-	251 40.9%	84 13.7%
Perceived knowledge							
I know what antibiotic resistance is	192 31.3%	342 55.8%	15 2.4%	12 2.0%	22 3.9%	8 1.3%	22 3.6%
I know what information to give to individuals about prudent use of antibiotics and antibiotic resistance	110 17.9%	390 63.6%	25 4.1%	9 1.5%	9 1.5%	67 10.9%	3 0.5%
I have sufficient knowledge about how to use antibiotics appropriately for my current practice	134 21.9%	391 63.8%	25 4.1%	8 1.3%	3 0.5%	51 8.3%	1 0.2%
Opportunity							
I have easy access to guidelines I need on managing infections	75 12.2%	305 49.8%	100 16.3%	19 3.1%	6 1.0%	103 17.0%	5 0.8%
I have easy access to the materials I need to give advice on prudent antibiotic use and antibiotic resistance	37 6.0%	246 40.1%	174 28.4%	28 4.6%	19 3.1%	106 17.3%	3 0.5%
I have good opportunities to provide advice on prudent antibiotic use to individuals	61 10.0%	351 57.3%	79 12.9%	13 2.1%	15 2.4%	92 15.0%	2 0.3%
Motivation to initiate antibiotic prescriptions							
I know there is a connection between my prescribing of antibiotics and emergence and spread of antibiotic resistant bacteria	122 19.9%	294 48.0%	58 9.5%	24 3.9%	22 3.6%	79 12.9%	14 2.3%
I am confident making antibiotic prescribing decisions	71 11.6%	391 63.8%	40 6.5%	9 1.5%	-	90 14.7%	12 2.0%
I have confidence in the antibiotic guidelines available to me	68 11.1%	398 64.9%	53 8.6%	4 0.7%	-	82 13.4%	8 1.3%
I have a key role in helping control antibiotic resistance	74 12.1 %	354 57.7%	50 8.2%	3 0.5%	-	127 20.7%	5 0.8%
I consider antibiotic resistance when treating a patient	116 18.9%	409 66.7%	21 3.4%	4 0.7%	-	59 9.6%	4 0.7%
I feel supported to not prescribe antibiotics when they are not necessary	130 21.2%	276 45.0%	120 19.6%	37 6.0%	-	44 7.2%	6 1.0%

Abbreviations: SA: strongly agree; A: agree; D: disagree; SD: strongly disagree; N/A: not applicable; U: undecided; IDU: I do not understand.

**Table 6:** The frequency with which prescribers provided antibiotics or resources related to prudent use of antibiotics, behavior on drivers for initiating prescriptions and antibiotic prescribing behaviors.

Item	>QD	>QW	NVR	R	QD	QW	N/A	IDR
Opportunity to provide antibiotics or resources related to prudent use of antibiotics								
How often did you prescribe antibiotics during the last one week?	169	107	76	53	86	86	24	12
	27.6%	17.5%	12.4%	8.7%	14.0%	14.0%	3.9%	2.0%
How often did you give out resources (e.g., leaflets or pamphlets) on prudent antibiotic use or management of infections to individuals during the last one week?	37	74	230	159	14	52	47	0
	6.0%	12.1%	37.5%	25.9%	2.3%	8.5%	7.7%	0.0%
How often did you give out advice related to prudent antibiotic use or management of infections to an individual during the last one week	143	115	69	86	39	103	19	39
	23.3%	18.8%	11.3%	14.0%	6.4%	16.8%	3.1%	6.4%
Behavior on drivers for initiating prescriptions								
How often would you have preferred not to prescribe an antibiotic but were not able to during the last one week?	86	88	124	132	33	98	0	52
	14.0%	14.4%	20.2%	21.5%	5.4%	16.0%	0.0%	8.5%
How often did the fear of patient deterioration or fear of complications lead you to prescribe antibiotics during the last one week?	53	67	132	157	34	118	0	52
	8.7%	10.9%	21.5%	25.6%	5.6%	19.3%	0.0%	8.5%
How often did you prescribe antibiotics because it took less time than to explain the reason why they are not indicated during the last one week?	47	42	219	154	35	63	0	53
	7.7%	6.9%	35.7%	25.1%	5.7%	10.3%	0.0%	8.7%
How often did you prescribe antibiotics in situations in which it is impossible for you to conduct a follow-up of the patient during the last one week	27	49	181	156	19	98	0	83
	4.4%	8.0%	29.5%	25.5%	3.1%	16.0%	0.0%	13.5%
How often did you prescribe an antibiotic to maintain the relationship with the patient during the last one week?	28	30	296	130	22	63	0	44
	4.6%	4.9%	48.3%	21.2%	3.6%	10.3%	0.0%	7.2%
How often did you prescribe an antibiotic because you were uncertain about the diagnosis of infection during the last one week	24	50	228	162	20	79	0	50
	3.9%	8.2%	37.2%	26.4%	3.7%	12.9%	0.0%	8.7%
Antibiotic prescribing behavior								
How often did you stop an antibiotic prescription earlier than the prescribed course length during the last one week?	15	28	274	138	15	61	0	82
	2.5%	4.6%	44.7%	22.5%	2.5%	10.0%	0.0%	13.4%
How often did you prescribe a shorter course of treatment as compared to available guidelines during the last one week?	20	33	254	148	13	57	0	88
	3.3%	5.4%	41.4%	24.1%	2.1%	9.3%	0.0%	14.4%
How often did you discontinue early (within three days after initiation) a treatment because bacterial infection was not likely after all during the last one week	20	33	249	159	15	54	0	83
	3.3%	5.4%	40.6%	25.9%	2.5%	8.8%	0.0%	13.5%

Abbreviations: QD: once a day; QW: once a week; NVR: never; R: rarely; N/A: not applicable; IDR: I do not remember.

## Data Availability

Data are available on reasonable request and in line with permission approval processes from the Jordan University of Science and Technology.

## Supplementary Materials

The following are available online at https://www.mdpi.com/article/10.3390/antibiotics10070858/s1, Questionnaire S1. Survey of physicians and dentists knowledge and attitudes about antibiotics and antibiotic resistance.
