# Supplementary material for: Prescribers’ Knowledge, Attitudes and Behaviors on Antibiotics, Antibiotic Use and Antibiotic Resistance in Jordan
Source: Antibiotics (Basel). 2021 Jul 15;10(7):858. doi: 10.3390/antibiotics10070858 (PMC8300611; doi:10.3390/antibiotics10070858)
Supplement: Supplementary file 1 [file antibiotics-10-00858-s001.zip › antibiotics-1289694-supplementary.pdf]

Appendix 1. Survey of physicians' and dentists' knowledge and attitudes about antibiotics and antibiotic resistance.

Dear Respondent,
You are invited to complete the following survey of physicians and dentists about their knowledge and attitudes about antibiotics and antibiotic resistance.
Researchers from UK, Jordan University of Science and Technology, and Yarmouk University are seeking responses from physicians and dentists. We would really value you completing the survey that will take 5 to 10 minutes to complete. It includes predominantly multiple-choice questions.
Please feel free to cascade the link of the survey widely to colleagues.

In which language would you prefer to complete this survey? بأيّ لغة تفضل اتمام الاستبيان ؟
English
Arabic

- **Demographic Section**

1. Please specify in which governorate you practice
Amman
Irbid
Salt
Zarqa
Madaba
Jerash
Ajloun
Mafraq
Karak
Tafilah
Ma'an
Aqaba

2. What is your core profession?
Medical doctor (e.g. general practice, surgeon, specialists, infectious disease physician)
Dentist

3. What is your predominant role? (i.e.>50% of your time)?
Generalist
Specialist
Academia/ Research

4. Where do you predominantly practice? (i.e. >50% of your time):
Hospital (any hospital type)
University (as an Academic) or research institute
Public clinic
Private clinic

5. **How many years have you been practicing in your current profession?**
0-2 years
3-5 years
6-10 years
11-15 years
16-20 years
21-25 years
>25 years

6. **What is your age?**
24-35 years
36-45 years
46-55 years
56-65 years
>66 years

7. **What gender do you identify with?**
Male
Female

➢ Knowledge about antibiotic use and antibiotic resistance

8. **Please answer whether you believe these statements are true or false.**

	True	False	Unsure
Antibiotics are effective against viruses			
Antibiotics are effective against cold infections			
Unnecessary use of antibiotics make them become ineffective			
Taking antibiotics has associated side effects or risks such as diarrhea, colitis, allergies			
Every person treated with antibiotics is at an increased risk of antibiotic resistant infection			
Antibiotic resistant bacteria can spread from person to person			
Healthy people can carry antibiotic resistant bacteria			
The use of antibiotics to stimulate growth in farm animals is legal in Jordan			

9. **At what level do you think it is most effective to tackle resistance to antibiotics? Select no more than 2.**
Individual level (prescribers)
Environmental/Animal Health
Regional/National Level
Global
Action at all levels needed
I do not know

10. **What strategies do you employ to prescribe antibiotics prudently? Select all that apply.**
Delayed prescribing/ back-up prescribing (delayed prescribing is a method whereby aprescription is issued by a health professional for use by the patient at a later date, if theirsymptoms do not improve)
Patient education
New patient consultation
None

Others: _______

11. How often do you prescribe antibiotics?
Everyday
Weekly
Monthly
Quarterly
Yearly

❖ ***For the next questions, to what extent do you agree or disagree with the following statements:***

12. "I know what antibiotic resistance is"
Strongly disagree
Disagree
Undecided
Agree
Strongly Agree
I do not understand the question
Not Applicable

13. "I know there is a connection between my prescribing of antibiotics and emergence and spread of antibiotic resistant bacteria":
Strongly disagree
Disagree
Undecided
Agree
Strongly Agree
I do not understand the question
Not Applicable

14. "I know what information to give to individuals about prudent use of antibiotics and antibiotic resistance":
Strongly disagree
Disagree
Undecided
Agree
Strongly Agree
I do not understand the question
Not Applicable

15. "I have sufficient knowledge about how to use antibiotics appropriately for my current practice":
Strongly disagree
Disagree
Undecided
Agree
Strongly Agree
I do not understand the question
Not Applicable

16. "I have a key role in helping control antibiotic resistance":
Strongly Disagree
Disagree
Undecided
Agree
Strongly Agree
I do not understand the question
Not applicable

17. "I have easy access to guidelines I need on managing infections":
Strongly Disagree
Disagree
Undecided
Agree
Strongly Agree
I do not understand the question
Not applicable

18. "I have easy access to the materials I need to give advice on prudent antibiotic use and antibiotic resistance":
Strongly Disagree
Disagree
Undecided
Agree
Strongly Agree
I do not understand the question
Not applicable

19. "I have good opportunities to provide advice on prudent antibiotic use to individuals":
Strongly disagree
Disagree
Neutral
Agree
Strongly agree
I do not understand the question

20. "Environmental factors such as wastewater in the environment are important in contributing to antibiotic resistance in bacteria from humans"?
Strongly disagree
Disagree
Neutral
Agree
Strongly agree
I do not understand the question

21. "Excessive use of antibiotics in livestock and food production is important incontributing to antibiotic resistance in bacteria from humans"?
Strongly Disagree
Disagree

Undecided
Agree
Strongly Agree
I do not understand the question

22. "I am confident making antibiotic prescribing decisions"?
Strongly Disagree
Disagree
Undecided
Agree
Strongly Agree
I do not understand the question

23. "I have confidence in the antibiotic guidelines available to me":
Strongly Disagree
Disagree
Undecided
Agree
Strongly Agree
I do not understand the question

24. " I consider antibiotic resistance when treating a patient ":
Strongly Disagree
Disagree
Undecided
Agree
Strongly Agree
I do not understand the question

25. I feel supported to not prescribe antibiotics when they are not necessary
Strongly Disagree
Disagree
Undecided
Agree
Strongly Agree
I do not understand the question

- ***Please answer the following questions, considering the last one week only in your clinical practice:***

26. How often did you prescribe antibiotics during the last one week?
Once a day
More than once a day
Once a week
More than once a week
rarely
never
I do not remember
Not Applicable

27. How often did you give out resources (e.g. leaflets or pamphlets) on prudent antibiotic use or management of infections to individuals during the last one week?

Once a day
More than once a day
Once a week
More than once a week
rarely
never
I do not remember
Not Applicable

28. How often did you give out advice related to prudent antibiotic use ormanagement of infections to an individual during the last one week?

Once a day
More than once a day
Once a week
More than once a week
Rarely
Never
I do not remember

29. How often would you have preferred not to prescribe an antibiotic but were not able during the last one week?

Once a day
More than once a day
Once a week
More than once a week
Rarely
Never
I do not remember

30. How often did the fear of patient deterioration or fear of complications lead you to prescribe antibiotics during the last one week?

Once a day
More than once a day
Once a week
More than once a week
Rarely
Never
I do not remember

31. How often did you prescribe antibiotics because it took less time than to explain the reason why they are not indicated during the last one week?

Once a day
More than once a day
Once a week
More than once a week
Rarely
Never

I do not remember

32. How often did you prescribe antibiotics in situations in which it is impossible for you to conduct a follow-up of the patient during the last one week?

Once a day
More than once a day
Once a week
More than once a week
Rarely
Never
I do not remember

33. How often did you stop an antibiotic prescription earlier than the prescribed course length during the last one week?

Once a day
More than once a day
Once a week
More than once a week
Rarely
Never
I do not remember

34. How often did you prescribe an antibiotic to maintain the relationship with the patient during the last one week?

Once a day
More than once a day
Once a week
More than once a week
Rarely
Never
I do not remember

35. How often did you prescribe an antibiotic because you were uncertain about the diagnosis of infection during the last one week?

Once a day
More than once a day
Once a week
More than once a week
Rarely
Never
I do not remember

36. How often did you prescribe a shorter course of treatment as compared to available guidelines during the last one week?

Once a day
More than once a day
Once a week
More than once a week
Rarely
Never

I do not remember

37. How often did you discontinue early (within three days after initiation) a treatment because bacterial infection was not likely after all during the last one week

Once a day
More than once a day
Once a week
More than once a week
Rarely
Never
I do not remember
